# Unusual Localization of *Hysterothylacium Incurvum* in *Xiphias gladius* (Linnaeus 1758) Caught in the Atlantic Ocean

**DOI:** 10.3390/pathogens11111315

**Published:** 2022-11-09

**Authors:** Giovanni De Benedetto, Ivan Corti, Renato Malandra, Kristian Riolo, Alessia Giannetto, Gabriella Gaglio

**Affiliations:** 1 Department of Veterinary Sciences, University of Messina, 98168 Messina, Italy; 2 Agenzia di Tutela della Salute dell’Insubria, 22100 Como, Italy; 3 Veterinario Responsabile S.S. Mercati Generali, ATS Città Metropolitana di Milano, 20137 Milano, Italy; 4 Department of Chemical, Biological, Pharmaceutical and Environmental Sciences, University of Messina, 98166 Messina, Italy

**Keywords:** swordfish, heart chambers, parasitic disease, Raphidascaridae, fish market

## Abstract

This study represents the first report of *Hysterothylacium incurvum* within swordfish (*Xiphias gladius*) heart chambers. Swordfish is a large pelagic teleost, considered one of the most appreciated fish worldwide. Among swordfish parasites, *Anisakis* sp. and *Hysterothylacium* sp. have been used to evaluate biological and ecological aspects of this teleost. Between 2021 and 2022, 364 *X. gladius* hearts, caught from the Atlantic Ocean (FAO 27.IXa and FAO 34 areas), were collected at the Milan fish market (Lombardy, Italy). Three specimens from FAO 27.IXa was positive for seven adult nematodes (*p* = 1.55%) within the heart chambers. Of these, three specimens were found within the bulbus arteriosus and 4 in the ventricle. All parasites were stored in 70% ethanol and processed for parasitological and molecular analysis using *Cox2*, *ITS regions/ITS-I-5.8S-ITS-II*, and rrnS genes. The analysis allowed us to identify the retrieved parasite as *H. incurvum*. According to our evaluation, the final localization is due to the movement of L3 larvae from the coelomic cavity to the bloodstream, with consequent development to the adult stage within the heart. Finally, the parasite localization, considered non-marketable fish parts, does not pose a significant risk to consumers, also considering the low zoonotic potential of *H. incurvum*.

## 1. Introduction

Swordfish (*Xiphias gladius*, Linnaeus 1758) is a large pelagic teleost characterized by a worldwide distribution, mainly in tropical and temperate areas, including the Mediterranean Sea. Despite its intense migratory aptitude, separate stocks, both in the Ocean and in the Mediterranean Sea, have been reported [1,2,3]. The high commercial value of swordfish caught from the Ocean and Mediterranean Sea has been reported [4]. Regarding oceanic swordfish populations, parasitic fauna associated with relative load has been described [5], confirming a significant division between the North and South Atlantic Ocean stocks [6]. Some genetic differences between Oceanic and Mediterranean *X. gladius* populations were reported [7]. Since 1990, genetic stock differentiation, and some stock movements, between Atlantic and Mediterranean Sea stocks have been reported [8]. Parasites have been used to identify various biological and ecological aspects of aquatic organisms, such as the integrity of food systems and indicators of marine ecosystem conditions, also providing significant data about global climatic changes [9]. Nematode larvae belonging to the genus *Anisakis* and *Hysterothylacium*, heterogeneous parasites characterized by a complex life cycle, were the most widely used as “biological tags” [10]. *Xiphias gladius* parasite fauna, such as crustaceans and trematodes, from the Indian and Pacific Oceans [11,12] and the Baltic Sea, have been reported [13]. Swordfish metazoan fauna sampled from the Mediterranean Sea [14,15] and the North Atlantic Ocean were described [3,16] and have been compared and reported. Among the metazoan parasites, *Hysterothylacium corrugatum*, *H. incurvum*, and *H. petteri* adult specimens were found in swordfish gastrointestinal tracts in the Mediterranean and Ocean areas [3,15]. Anisakidae larvae, genetically identified as *Anisakis pegreffii* and *A. physeteris*, were reported in the Mediterranean Sea [15]. *A. simplex* (sensu strictu), *A. paggiae*, *A. brevispiculata*, and *A. physeteris* larvae were found and molecularly identified in *X. gladius* celomic organs serosae, caught off the Portuguese Atlantic Ocean areas [3]. The copepod *Pennella instructa*, attached to the skin [16] and up to the heart chambers [17], has been described worldwide. *Contracaecum* sp. larvae, generally found in the teleost body cavity [18], pericardial sac [19], and celomic organs serosa [20], were found and histologically described in the atrium and ventricle heart chambers of the freshwater species, fathead minnows (*Pimephales promelas*) and nine-spined stickleback (*Pungitius pungitius*) caught from High Rock Lake (Nord Carolina, USA) [21]. After a thorough evaluation of data reported in the literature, in which the presence of adult nematodes was reported only in the gastrointestinal lumen, the present study aims to document the unusual localization of adult nematodes inside the hearth chamber of swordfish caught in the Atlantic Ocean.

## 2. Materials and Methods

### 2.1. Sample Collection and Parasitological Assessment

From February 2021 to May 2022, 364 hearts of *X. gladius* were collected during official veterinarian checks at the Milan fish market (Milan, Lombardia, Italy). All examined specimens were caught using hooks and lines fishing methods. In total, 193 fish were caught in the Atlantic, Northeast, Portuguese Waters East Area (FAO 27.IXa), while 171 were caught in the Atlantic, Eastern Central Area (FAO 34). After an external examination, all fish hearts were opened for routine official veterinary activity. Biological indices of body weight (BW) and total length (TL) were recorded for each specimen, and the mean weight (MW) and mean length (ML) were calculated. All retrieved parasites were immediately stored in 70% ethanol and transferred to the laboratory of Parasitology and Parasitic Diseases, University of Messina, for subsequent examinations, where all samples were divided into two stocks, identified as Area 1 (Northeast, Portuguese Waters East Area) and Area 2 (Atlantic, Eastern Central). *Xiphias gladius* specimens sampled from Area 1 had an MW of 50.5 kg and an ML of 160.1 cm, while specimens from Area 2 had an MW of 39.1 kg and an ML of 155.3 cm. Morphological evaluation was performed with an optic stereo microscope (SteREO Discovery.V12 Zeiss, Jena, Germany) following the keys suggested by Bruce and Cannon [22], and all pictures were taken with a digital camera system (Axiocam Mrc, Axiovision, Zeiss, Jena, Germany). Epidemiological indices of prevalence (P%), mean abundance (MA) and mean intensity (MI) was estimated following the technique reported by Bush et al. [23].

### 2.2. Molecular Analysis

#### 2.2.1. DNA Extraction from Parasites 

Genomic DNA extraction from parasites was performed using the Nucleo Spin Plant II kit (Macherey-Nagel, Düren, North Rhine-Westphalia, Germany), according to the manufacturer’s instructions. NanoDrop 2000 (Thermo Scientific; Wilmington, MA, USA) was used to measure UV absorbance at 260, 280, and 230 nm to verify DNA quantity and purity. *Nuclear ribosomal ITS regions (ITS-I-5.8S-ITS-II), a small subunit of the mitochondrial ribosomal RNA gene* (*rrnS*), and *cytochrome C oxidase subunit II (cox2)* were used as phylogenetic markers in the polymerase chain reaction (PCR). 

#### 2.2.2. Polymerase Chain Reaction and Sequence Analysis

PCR was performed using 500 ng of genomic DNA and Taq DNA Polymerase Recombinant kit (Invitrogen, Carlsbad, California, United States) in a 50 µL reaction volume using the Ep-Gradient Mastercycler (Eppendorf, Hamburg, Germany). For the *nuclear ribosomal ITS region* amplifications, the following PCR conditions were used: after the first step of 95 °C for 10 min, DNA was subjected to 35 cycles of 95 °C for 30 s, 52 °C for 40 s, and 72 °C for 75 s, with a final extension of 72 °C for 7 min. For the *small subunit of the mitochondrial ribosomal RNA gene* amplification, the cycling was as follows: denaturing at 95 °C for 10 min followed by 40 cycles of 95 °C for 30 s, 55 °C for 30 s, and 72 °C for 30 s with an initial denaturation of 95 °C for 10 min and a final extension of 72 °C for 7 min. The *Cytochrome C oxidase subunit II* was amplified, performing 35 cycles of 95 °C for 30 s, 52 °C for 40 s, and 72 °C for 75 s. 

PCR products were resolved by 1.5% agarose gel electrophoresis to verify product size; the fragments were then purified using the E.Z.N.A Gel Extraction Kit (OMEGA, Omega Bio Tek, Norcross, GA, USA), following the manufacturer’s protocol. DNA sequencing of the purified fragments was performed in both forward and reverse directions on the Applied Biosystems 3730 DNA Analyzer (Thermo Fisher Scientific, Waltham, MA, USA), using the same primers used for amplification (Table 1).

The DNA sequences obtained from the isolates (XG1-2022) were analyzed by BLASTN similarity search against the National Center for Biotechnology Information (NCBI; https://blast.ncbi.nlm.nih.gov/Blast.cgi, accessed on 12 September 2022) database to calculate the statistical significance of the matches, and alignments were performed using the ClustalW algorithm (https://www.genome.jp/tools-bin/clustalw, accessed on 13 September 2022).

Phylogenetic analyses were performed using MEGA X [24], and Maximum likelihood (ML) trees were constructed by selecting the GTR + G + I nucleotide substitution model with the bootstrap method (1000 replications).

**Table 1:** List of the primers used in this study.

Gene	Forward Primer Sequence	Reverse Primer Sequence	Size (bp)	Reference
***ITS*** * regions*	GTAGGTGAACCTGCGGAAGGATCATT	TTAGTTTCTTTTCCTCCGCT	900	Pekmezci, G.Z., Yardimci, B. [25]
***rrnS***	TTGTTCCAG AATAATCGGCTAGACTT	TCTACTTTACTACAACTTACTCC	530	D’Amelio et al. [26]
***cox2***	TTTCTAGTTATATAGATTGRTTYAT	CACCAACTCTTAAAATTATC	629	Quiazon et al. [27]

## 3. Results

**Figure 1:**
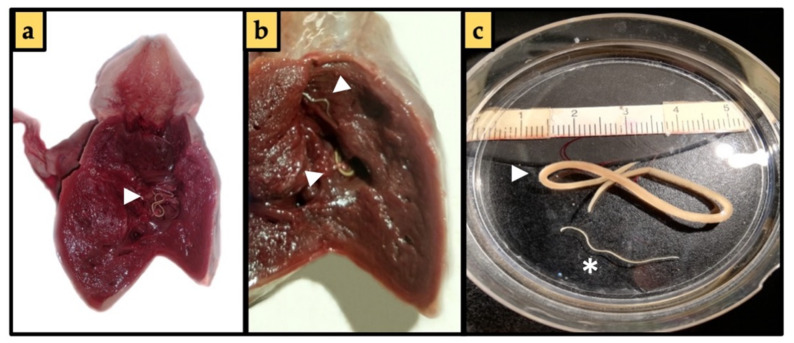
*Hysterothylacium incurvum* specimens (**a**), *H. incurvum* inside the ventricles of *Xyphias gladius* heart (arrowhead) (**b**), *H. incurvum* specimens between bulbus arteriosus and ventricles of *X. gladius* heart (arrowhead) (**c**), *H. incurvum* male (asterisk) and female (arrowhead) under a stereomicroscope.

Three of the 171 specimens caught from Area 1 were positive for the presence of adult nematodes inside the hearth chambers (*n* = 7; *p* = 1.55%, MA = 0.04, MI = 2.33); of these, three specimens were found inside the bulbus arteriosus and four specimens in the ventricle (Figure 1); 3 of the nematodes were males (2.5 up to 3.7 cm), and four females (6 up to 11 cm). The morphological characteristic of the retrieved parasites allowed us to identify them as *Hysterothylacium* sp.

### Molecular Identification of Hysterothylacium *sp.*

All specimens showed positive amplification for *ITS regions*, *rrnS*, and *cox2* genes. The nucleotide sequences of the amplified products of each gene were identical among biological replicates. The representative DNA sequences for *ITS regions*, *rrnS*, and *cox2* were submitted to GenBank (accession numbers *ITS*: OP675472, *rrnS*: OP675473, and *cox2*: OP675471, respectively). The representative sequences of *ITS regions* showed 98.27% similarity to *Hysterothylacium* sp. (MT365536.1, E value 0.0 and query cover 90%) with 7 nt of difference. The *rrnS* sequences showed 90.76% similarity to *Hysterothylacium* sp. (MF140352.1, E value 2E-154 and query cover 93%) with 39 nt of difference. The obtained sequences of *cox2* showed 97% similarity to *H. incurvum* (MW456073.1, E value 0.0, and query cover 92%) with 18 nt of difference. These findings indicated that no *ITS* and *rrnS* sequences from *H. incurvum* were available in GenBank to date. 

**Figure 2:**
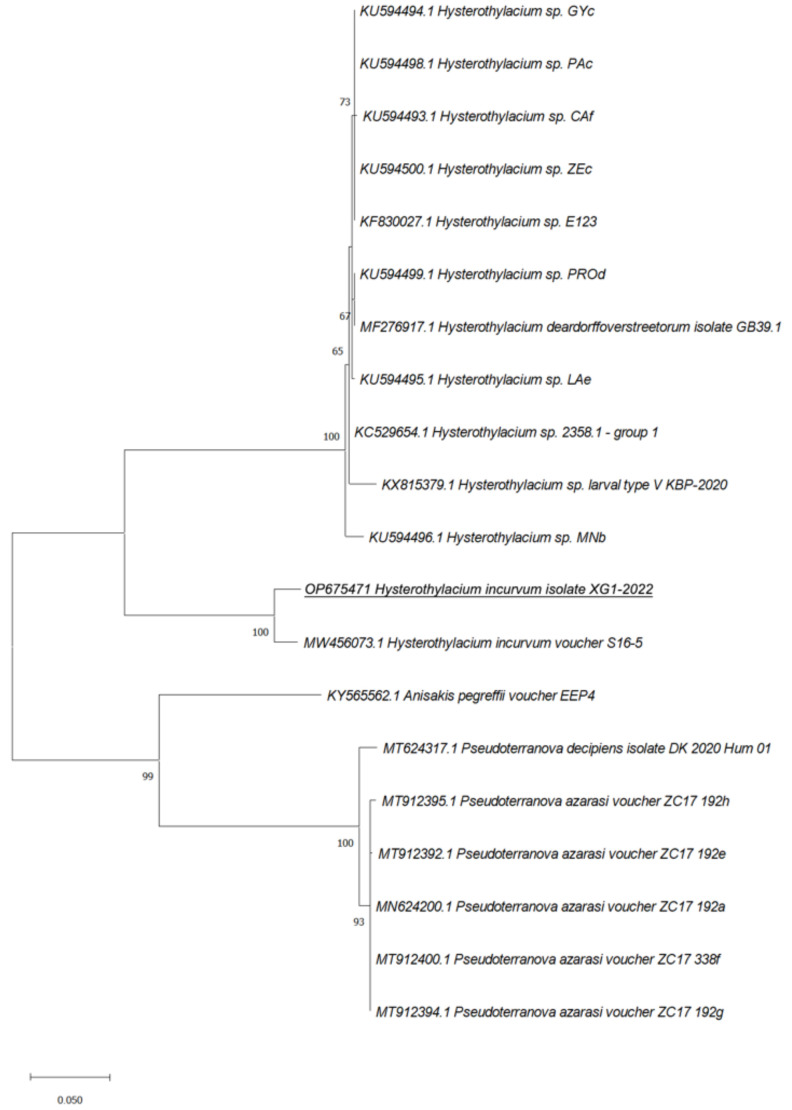
Phylogenetic relationships between the isolates of the present study and other Ascaridoidea as inferred from sequences of *cox2* analyzed by Maximum-likelihood. Only bootstrap values above 60 are shown. GenBank accession numbers are indicated before species names. The species analyzed in this study are underlined.

**Figure 3:**
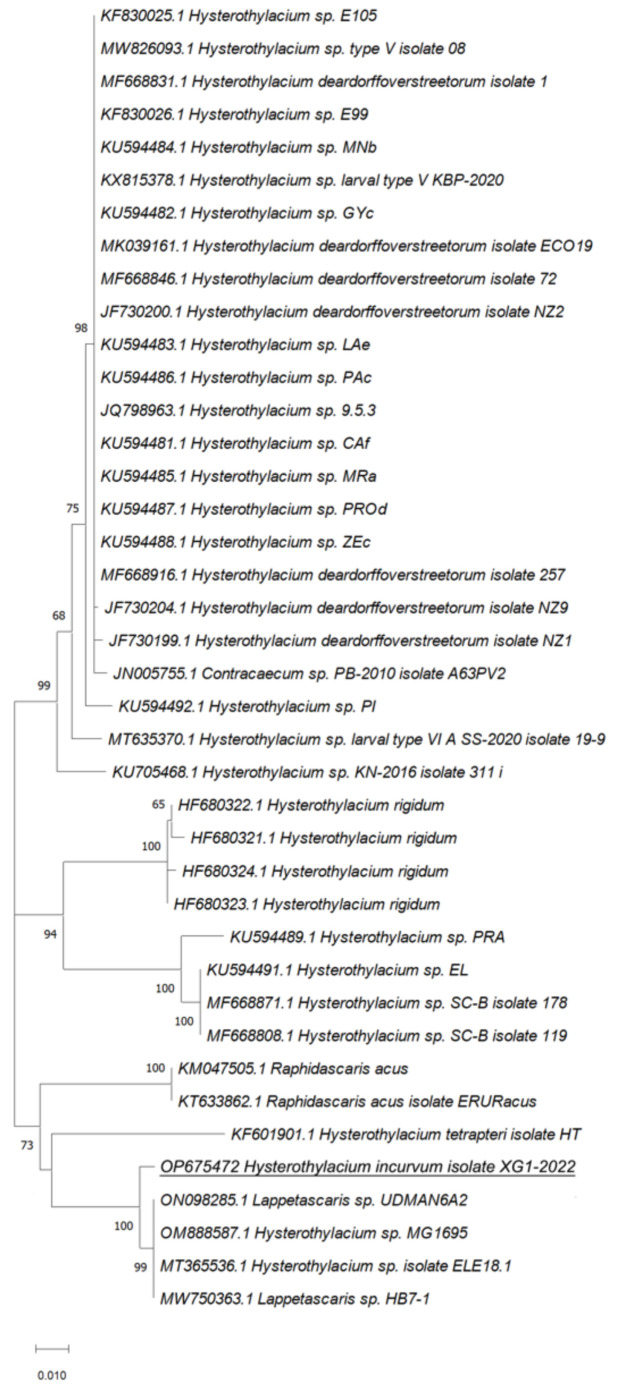
Phylogenetic relationships between the isolates of the present study and other Ascaridoidea as inferred from sequences of *ITS* analyzed by Maximum-likelihood. Only bootstrap values above 60 are shown. GenBank accession numbers are indicated before species names. The species analyzed in this study are underlined.

**Figure 4:**
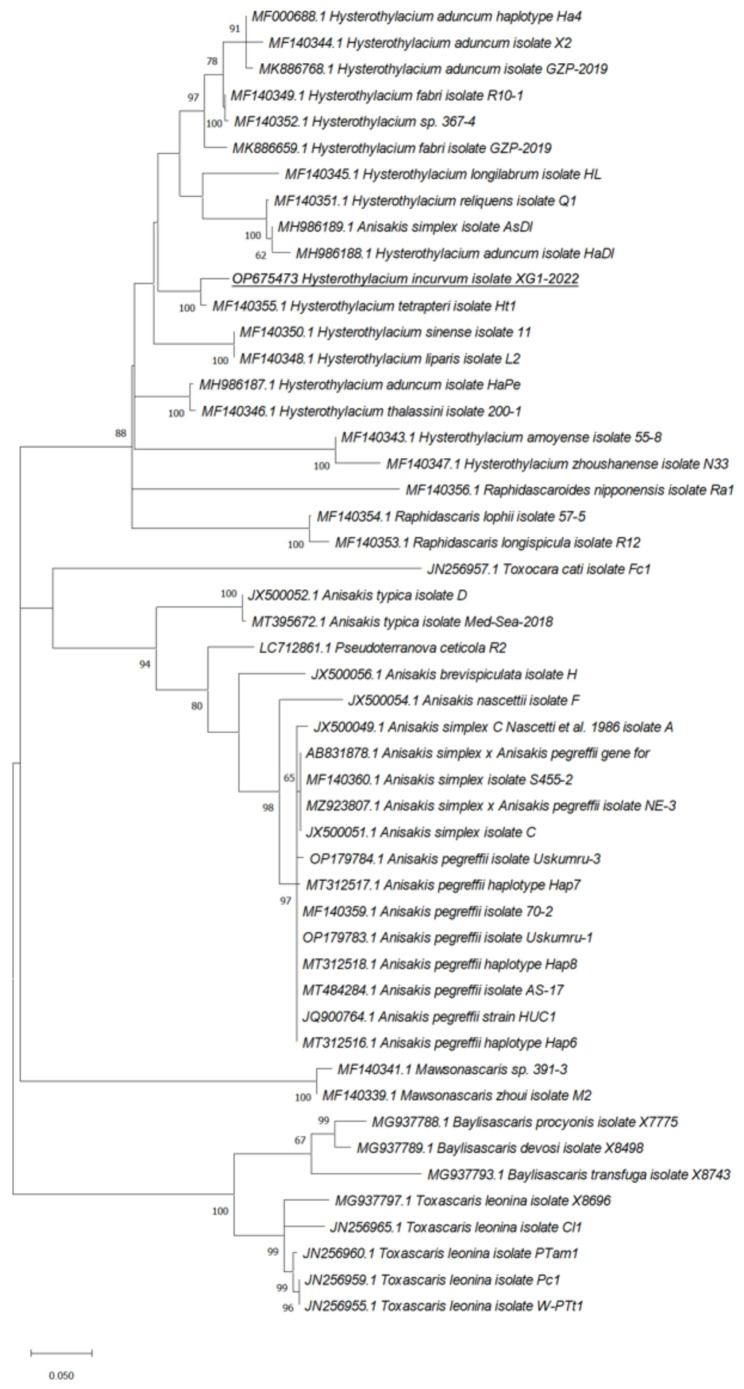
Phylogenetic relationships between the isolates of the present study and other Ascaridoidea as inferred from sequences of *rrnS* analyzed by Maximum-likelihood. Only bootstrap values above 60 are shown. GenBank accession numbers are indicated before species names. The species analyzed in this study are underlined.

Phylogenetic analyses of our sequences with the relative *ITS*, *rrnS*, and *cox2* sequences from Ascaridoidea previously deposited in GenBank showed that the *cox2* marker was the most effective in the species identification as sequences from our isolates were in the same clade with *H. incurvum* (MW456073.1) supported by a value of 100 at the node and in a separate branch including all the *cox2* sequences from *Hysterothylacium* sp. retrieved from GenBank (Figure 2, Figure 3 and Figure 4). 

## 4. Discussion

The present study represents the first report of *Hysterothylacium incurvum* in the heart chambers of *X. gladius*, considered one of the most appreciated fish species worldwide.

*Hysterothylacium* sp. represents one of the most isolated parasites in swordfish [3,28,29,30]; our molecular evaluation, compared to other *Hysterothylacium* sp. sequences reported by Garcia et al. [3], allowed us to identify all the specimens as *H. incurvum*, adding significant information about the species that parasitize the swordfish in the studied area.

The notable finding of adult *Hysterothylacium* inside the heart chambers of *X. gladius* highlights a characteristic parasite adaptation against the high blood pressure present in the infection site. The only finding of *Contracaecum* sp. larvae inside the heart chambers of freshwater fish [21] did not show any host’ inflammatory response. In the present study, none of the positive specimens showed a reduction in body weight, suggesting a complete host-parasite adaptation. Furthermore, the used fishing technique does not show any reduction of predatory activities, characteristic of *X. gladius*. According to Kabata [31], there is the possibility that during the early stage, in the present study L3 larvae, during the physiological intra vitam movement between the celomic cavity and muscle tissue, the parasite can move into the ventral aorta, reaching the bulbus arteriosus and ventricle of the heart, causing bloodstream occlusion, in the case of massive infection. In large healthy fish, there is the possibility of a natural adaptation of the heart chambers, able to modify their structure against occlusive injuries [31,32]. In the case reported here, no macroscopically appreciable structural adaptation was observed, probably due to the different parasite size and body compared to the aforementioned cases. Usually, *Hysterothylacium* sp. larvae have been used to identify fish stock between the Ocean and the Mediterranean Sea [15]. Our study confirmed ocean stock heterogeneity, adding information on body distribution and possible intra vitam migration of *Hysterothylacium* sp. larvae in *X. gladius*. According to Kabata [31], only during a massive infection could the presence of parasites create tissue damage, followed by host physiological adaptations, as also reported by Schuurmans Stekhoven [32]. The low parasitic load per specimen described in the present study, also considering the huge caliber of the ventral aorta and the size of the heart chambers, suggested a partial larval migration from the coelomic cavity to the bloodstream. Furthermore, the mixed infections described by Kabata [31] as an additional cause of occlusive damage in the heart chambers cannot be considered in the present study; indeed, morphological evaluation, associated with molecular analysis, allowed us to identify the found parasite as *H. incurvum*. The swordfish heart involvement during parasite infection previously reported [17] was significantly different from our case, as *Pennella instructa* involves heart tissues after skin and muscle penetration; in our case, we can speculate that the adult *H. incurvum* developed in the bloodstream and heart chambers, after a L3 larvae penetration in other body districts. Among the three phylogenetic markers analyzed in this study, the *cox2* gene was the most suitable marker for identifying *H. incurvum* in *X. gladius*, thus contributing to the morphological characterization of these parasites in fish. The multi-locus approach would not have been effective as no *ITS regions*, and *rrnS* sequences from *H. incurvum* were deposited in GenBank to date; therefore, *ITS* and *rrnS* sequences obtained in this study can provide new molecular markers for the identification of *H. incurvum* in future studies.

## 5. Conclusions

The present study improves the parasitological knowledge of the host/parasite relationship between *H. incurvum* and *X. gladius*. In particular, this paper provides an update on the parasite localization and development stage of this parasite in swordfish. The observed localization of *H. incurvum* that involve nonedible and non-marketable parts may represent a negligible risk for the consumers, also considering the low zoonotic potential of this parasite [33].

## Data Availability

The data presented in this study are available on request from the corresponding author.
